# Cognitive Impact of Colorectal Cancer Surgery in Elderly Patients: A Narrative Review

**DOI:** 10.3390/cancers18030417

**Published:** 2026-01-28

**Authors:** Oswaldo Moraes Filho, Bruno Augusto Alves Martins, Tuane Colles, Romulo Medeiros de Almeida, João Batista de Sousa

**Affiliations:** 1 Coloproctology Service, University Hospital of Brasília, University of Brasilia, Brasília 70830-200, DF, Brazil; bruno.augusto@ebserh.gov.br (B.A.A.M.); tuane.colles@ebserh.gov.br (T.C.); romulo.almeida@ebserh.gov.br (R.M.d.A.); sousajb@unb.br (J.B.d.S.); 2 SHIN Qi 8 Conjunto 3 casa 13, Lago Norte, Brasília 71520-230, DF, Brazil; 3 School of Medicine, University of Brasília, Brasília 70910-900, DF, Brazil

**Keywords:** postoperative cognitive dysfunction, POCD, delirium, colorectal cancer, elderly, geriatric surgery, Enhanced Recovery After Surgery, ERAS, neuroinflammation, perioperative neurocognitive disorders

## Abstract

Background/Objectives: Postoperative cognitive dysfunction (POCD) represents a significant and potentially preventable complication in elderly patients undergoing colorectal cancer surgery, with reported incidence ranging from 2.8% to 62.2% depending on perioperative management strategies and assessment methods. This narrative review synthesizes current evidence on the epidemiology, pathophysiology, risk factors, and prevention strategies for POCD in this vulnerable population. Methods: A comprehensive narrative review was conducted to examine the current literature on POCD in elderly colorectal cancer patients. Evidence was synthesized from published studies addressing epidemiology, assessment tools, risk factors, pathophysiological mechanisms, and prevention strategies, with a particular focus on Enhanced Recovery After Surgery (ERAS) protocols and multicomponent interventions. Results: Advanced age, pre-existing cognitive impairment, frailty, and surgical complexity emerge as key risk factors for POCD. ERAS protocols demonstrate substantial protective effects, reducing POCD incidence from 35% under conventional care to as low as 2.8% in optimized pathways. The pathophysiology involves multifactorial mechanisms, including neuroinflammation, blood–brain barrier disruption, neurotransmitter dysregulation, and oxidative stress, with surgical trauma triggering systemic inflammatory cascades that activate microglial responses within the central nervous system. Evidence-based prevention strategies include preoperative cognitive and frailty screening, minimally invasive surgical techniques, multimodal opioid-sparing analgesia, regional anesthesia, depth-of-anesthesia monitoring, and structured postoperative care bundles adapted from the Hospital Elder Life Program. Conclusions: The integration of comprehensive perioperative cognitive care protocols represents a critical priority as surgical volumes in elderly populations continue to expand globally. Emerging directions include biomarker development for early detection and risk stratification, precision medicine approaches targeting individual vulnerability profiles, and novel therapeutic interventions addressing neuroinflammatory pathways. Standardized assessment tools, multidisciplinary collaboration, and implementation of evidence-based preventive interventions offer substantial promise for preserving cognitive function and improving long-term quality of life in elderly colorectal cancer patients.

## Simple Summary

As the global population ages, more elderly patients are undergoing colorectal cancer surgery. However, many of these patients experience cognitive problems after surgery, such as confusion or memory difficulties, which can significantly affect their recovery and quality of life. This review examines why these cognitive complications occur in older adults after colorectal cancer surgery and identifies who is most at risk. We found that age, pre-existing cognitive problems, frailty, and the complexity of the surgery are major risk factors. The review also explores how inflammation in the brain and disruption of the blood–brain barrier contribute to these problems. Importantly, we highlight evidence-based strategies for preventing cognitive decline, including optimized surgical protocols and specialized care programs for elderly patients. Understanding these factors can help doctors better identify high-risk patients and implement protective measures, ultimately improving outcomes and preserving cognitive function in older adults undergoing cancer surgery.

## 1. Introduction

Colorectal cancer (CRC) is among the most prevalent malignancies globally, with a particularly high incidence in individuals aged 65 years and older [1]. As the global population ages, the number of elderly patients requiring surgical resection—the primary curative intervention—will increase [2,3]. The perioperative management of older adults poses unique challenges beyond standard oncological care. Notably, the effect of major surgery on cognitive function has emerged as a significant and frequently underrecognized complication, carrying substantial consequences for this vulnerable demographic.

Postoperative cognitive dysfunction (POCD) encompasses a range of neurocognitive impairments that may arise after surgery and anesthesia [4]. POCD should be distinguished from postoperative delirium, an acute and fluctuating confusional state [5,6]. Although delirium is typically transient, it constitutes a significant risk factor for the subsequent development of persistent POCD, which can endure for weeks or months and substantially diminish long-term functional independence and quality of life [7,8]. The incidence of POCD in elderly patients following colorectal surgery is considerable, with studies reporting rates between 14% and over 30%, highlighting the significance of this complication [9,10,11].

The etiology of POCD is multifactorial, arising from the interplay between patient-specific vulnerabilities and perioperative stressors. Advanced age, baseline cognitive impairment, and frailty are consistently recognized as the most significant predisposing factors, diminishing physiological and cognitive reserves [5,9,12]. These vulnerabilities are further exacerbated by surgical trauma and the associated systemic inflammatory response, both of which are central to the neuroinflammatory processes underlying POCD pathophysiology [6,13,14].

With the rising number of elderly patients undergoing colorectal cancer surgery, elucidating the risk factors, pathophysiology, and effective prevention and management strategies for POCD has become a clinical and public health imperative [7,15]. This narrative review synthesizes current evidence on the epidemiology, risk factors, pathophysiological mechanisms, and prevention strategies for POCD in elderly CRC patients, to inform perioperative care and identify critical gaps for future research.

## 2. Methods

This narrative review synthesizes current evidence on postoperative cognitive dysfunction in elderly patients undergoing colorectal cancer surgery. A comprehensive literature search was conducted across multiple databases, including PubMed, Cochrane Library, and Web of Science, using relevant search terms for postoperative cognitive dysfunction, colorectal cancer, elderly patients, and surgical outcomes.

The search strategy employed both Medical Subject Headings (MeSH) terms and free-text keywords, including combinations of “postoperative cognitive dysfunction”, “POCD”, “delirium”, “colorectal surgery”, “elderly”, “geriatric”, and “cognitive impairment”. Over 130 relevant studies were identified and analyzed, including observational studies, randomized controlled trials, systematic reviews, and meta-analyses published in peer-reviewed journals. No formal meta-analysis was performed; instead, qualitative integration of evidence was used to provide a comprehensive overview tailored to elderly colorectal cancer patients.

## 3. Epidemiology and Assessment Tools

### 3.1. Incidence and Prevalence Patterns

#### 3.1.1. Incidence by Surgical Context

The reported rate of postoperative cognitive dysfunction (POCD) or its acute presentation, postoperative delirium (POD), ranges greatly in the literature due to the dissimilarity of investigated populations, types of surgeries performed, definitions applied, and perioperative management policies. A key systematic review and meta-analysis by Yang et al. (2020), which specifically studied patients undergoing CRC surgery, found a pooled POCD incidence of 14% [9]. In the same line, a recent comprehensive meta-analysis by Huang et al. (2025), which examined postoperative neurocognitive disorders among non-cardiac surgery patients, found similar prevalence patterns and risk factors, thereby reinforcing the generalizability of findings in colorectal cancer surgery [16].

The incidence of postoperative delirium in elderly patients after elective colorectal surgery varies greatly. A remarkable randomized controlled trial by Jia et al. (2014) found delirium rates of 3.4% in patients managed with a fast-track protocol compared to 12.9% in those receiving traditional care, highlighting the significant impact of perioperative care on cognitive outcomes [17]. A prospective cohort study by Raats et al. (2015) reported an overall delirium incidence of 21% in elderly patients undergoing colorectal surgery [10]. Additional studies have documented similar ranges, with Wu et al. (2016) finding 26.4% POCD incidence [18], while Chew et al. (2022) in their systematic review and meta-analysis highlighted the persistent challenge of postoperative delirium in elderly surgical patients despite various monitoring strategies [19]. Notably, Mangnall et al. (2011) showed a high incidence of 35% POD in 118 elderly patients (mean age 71.8 years) undergoing colorectal cancer surgery, with age, male sex, and ICU admission being significant risk factors [20].

The context of surgical urgency may significantly influence the incidence of delirium. POD incidence is substantially higher in emergency colorectal surgery compared to elective procedures. An extensive retrospective cohort study by Lander et al. (2025) of over 5.5 million older adults undergoing non-cardiac surgery found that emergency surgery was associated with a 3.4-fold higher incidence of postoperative delirium compared to elective surgery (7.5% vs. 2.2%) [21]. In colorectal surgery specifically, Desrochers et al. (2022) demonstrated that emergency surgery was independently associated with higher delirium rates and that patients experiencing delirium had a 140-fold higher mortality rate (14% vs. 0.1%) compared to those without delirium [22]. All of these findings indicate that emergency surgery is a significant risk factor for POCD.

#### 3.1.2. Impact of Baseline Cognitive Status

Some baseline conditions can make a difference in which elderly patients are more susceptible to POCD. Advanced age is one of the most consistently reported risk factors. A meta-analysis by Yang et al. (2020) specific to colorectal cancer surgery confirmed that age of 65 or 70 years or older is a significant predictor of POCD [9]. Similarly, a meta-analysis by Scholz et al. (2016) in a broader cohort of elderly patients undergoing major gastrointestinal surgery also identified advanced age as an important risk factor [5,23]. Furthermore, previous baseline cognitive reality is a critical determinant of risk. The same meta-analysis by Yang et al. also found that a preoperative Mini-Mental State Examination (MMSE) score below 24 was strongly associated with an increased risk of POCD [9]. This is corroborated by cohort studies in colorectal surgery, such as the one by Hewitt et al. (2018), which reported that 41% of patients with pre-existing cognitive impairment developed postoperative delirium, compared to only 11% of those with normal baseline scores [11]. Other patient-related determinants are identified as significant predictors of POCD, in addition to age and baseline cognition. Cui et al. (2025) demonstrated that lower educational level, poor sleep quality, and higher postoperative pain intensity were independent risk factors for POD in elderly colorectal cancer patients [24].

The marked heterogeneity in the reported incidence of POCD or delirium, ranging from 3.4%, with optimized fast-track protocols, to up to 35%, in high-risk populations, reflects the multifactorial nature of these complications and highlights relevant differences in patient characteristics, surgical interventions, and perioperative care modalities, as well as in definitions of cognitive decline. The significant drop in delirium rates observed with the enhanced recovery protocols (from 12.9% to 3.4% in Jia) also illustrates that this is a modifiable complication. It highlights the critical importance of evidence-based perioperative care pathways. Emergency surgery demonstrates higher delirium rates than elective procedures, emphasizing the compounding effect of acute physiological stress. These epidemiological patterns show that POCD risk stratification must account for both patient-specific factors (age, baseline cognition, comorbidities) and procedure-related variables (surgical urgency, perioperative care pathway, surgical complexity). The identification of high-risk patients through comprehensive preoperative assessment is therefore essential for implementing targeted preventive interventions and optimizing perioperative care.

### 3.2. Assessment Tools

#### 3.2.1. Preoperative Cognitive Screening Instruments

The assessment of POCD is complicated by the lack of a single, universally accepted diagnostic framework, particularly in the busy perioperative setting. Although complete neuropsychological testing represents the gold standard, its resource demands limit its routine application in clinical practice [4,25]. To this end, there has been a trend toward practical screening methods. The Mini-Mental State Examination (MMSE) and the Montreal Cognitive Assessment (MoCA) are commonly used, though they may have limited sensitivity for subtle cognitive changes [12]. More recently, simpler tools have been validated for this specific population. A prospective study by Fiamanya et al. (2022) in a cohort of elective colorectal surgery patients demonstrated the feasibility of using the brief Mini-Cog screening tool and the significant association between an abnormal Mini-Cog result and postoperative delirium [25]. Another study, Hattori et al. (2009), demonstrated the utility of the E-PASS (Estimation of Physiologic Ability and Surgical Stress) system combined with the NEECHAM Confusion Scale for predicting POD in elderly patients undergoing digestive surgery (56% colorectal), reporting a delirium incidence of 54.7% and establishing these tools as effective screening instruments [26].

#### 3.2.2. Frailty Assessment Tools

Beyond cognitive-screening instruments, new assessment approaches have been developed. Wang et al. (2025) reported that preoperative quantitative ultrasound of the quadriceps muscle had high accuracy (AUC 0.966) for predicting POCD in elderly patients undergoing gastrointestinal surgery [27]. Furthermore, comprehensive geriatric assessment incorporating multiple domains (IADL, MMSE, visual impairment, depression screening) might also have good sensitivity and specificity in predicting POCD [28]. The utility of comprehensive geriatric assessment (CGA) for predicting postoperative complications in elderly colorectal cancer patients was validated by Mokutani et al. (2016) in 156 patients aged ≥ 75 years, demonstrating that MMSE was a significant independent predictor of both overall complications and delirium [29]. Ketelaers et al. (2023) validated the G8 screening tool in 170 elderly CRC patients: patients with a G8 test score ≤ 14 were at significantly greater risk for POCD (7.7%) than those with scores > 14 (1.1%), indicating that frailty assessment should be included in routine preoperative evaluation [30].

A stepwise approach, employing the Identification of Seniors at Risk (ISAR) questionnaire as an initial screen and a more comprehensive geriatric assessment for elderly patients identified as high-risk, has been shown to enable risk stratification and targeted prehabilitation. Indrakusuma et al. (2015) also found that elderly patients with colorectal cancer receiving such a comprehensive review, including cognitive assessment (MMSE), nutritional assessment (Mini Nutritional Assessment), and depression screening (GDS), had significantly reduced lengths of hospital stay compared to those who received routine care, with targeted interventions provided for nutritional supplementation and prophylactic haloperidol in individuals at higher risk of delirium development [31].

#### 3.2.3. Practical Implementation Strategies

While there are an increasing number of assessment instruments available, the practical application must be influenced by feasibility and proven outcomes. For routine cognitive screening in elderly colorectal cancer patients, the Mini-Cog offers the best balance of brevity (3–5 min), simplicity, and predictive validity for postoperative delirium. Institutions with access to geriatric services should consider a two-step approach: initial screening with ISAR or G8 to identify high-risk patients, followed by a comprehensive geriatric assessment (including MMSE, nutritional evaluation, and depression screening) for those who screen positive. This approach supports cost-effective risk classification and planned preoperative optimization. While emerging technologies such as quantitative muscle ultrasound demonstrate impressive predictive accuracy, their requirement for specialized equipment currently limits widespread adoption. The key to successful implementation is not selecting the most sophisticated tool, but rather that an assessment approach can actually be implemented reliably, given the difficulties of local resources and workflow, paired with defined intervention pathways based on one’s use of assessment data.

Table 1 provides a comprehensive summary of validated assessment tools for POCD risk stratification and delirium detection in elderly colorectal cancer patients, including their clinical utility, predictive accuracy, and practical implementation considerations.

## 4. Risk Factors

### 4.1. Patient-Related Risk Factors

There are many patient-related factors associated with POCD in patients undergoing colorectal cancer operation that can generally be categorized into demographic/clinical, functional/nutritional status, and biological/imaging markers.

#### 4.1.1. Age, Cognitive Status, and Comorbidities

Age, poor preoperative cognitive function, and more comorbidities seem to be the demographic and clinical factors with the most consistent and robust evidence. The pivotal meta-analysis by Yang et al. (2020) identified age older than 65 years, a Mini-Mental State Examination (MMSE) score below 24, and an American Society of Anesthesiologists (ASA) physical status classification greater than II as key independent risk factors [9]. These results are in line with a second meta-analysis on major gastrointestinal surgery published by Scholz et al. (2016), and substantiated by a large prospective cohort study of Janssen et al. (2019) which found cognitive impairment (OR 4.1), ASA classification greater than or equal to 3 (OR 2.0), and the colorectal cancer diagnosis itself (OR 4.0) to be independent risk factors for POCD [5,32]. In addition, and interestingly, specific comorbidities such as hyperlipidemia have been demonstrated to enhance POD risk for this population [33].

#### 4.1.2. Frailty and Nutritional Status

In addition, frailty and malnutrition have been identified as critical predictors of vulnerability. Frailty, a condition of susceptibility to stressors, is highly correlated with poor postoperative outcomes such as delirium and decreased long-term functional status [12,34,35]. This has also been applied to “cognitive frailty” (comprising both cognitive impairment and physical frailty), demonstrating its substantial predictive value for postoperative complications [36]. Sarcopenia (a decline in muscle mass) and a decrease in physical activity, as measured by handgrip strength, have also been shown to be independent predictors of postoperative delirium [37,38]. Nutritional status is closely linked to frailty, with low serum albumin levels being a consistent risk factor [24]. The Geriatric Nutritional Risk Index (GNRI), a practical and straightforward nutritional screening tool, has also been validated as a predictor of postoperative complications, including delirium, in elderly colorectal cancer patients [39]. The critical importance of this interplay among frailty, nutrition, and cognitive outcomes is highlighted from an oncogeriatric perspective, underscoring the need for comprehensive geriatric assessment to optimize perioperative care [40].

#### 4.1.3. Biological and Imaging Markers

Finally, emerging evidence suggests that underlying biological and structural brain changes can serve as important vulnerability markers. Preoperative cerebral lacunar infarcts detected on neuroimaging have been identified as significant predictors of POD in elderly patients undergoing major abdominal surgery, suggesting that pre-existing cerebrovascular disease increases susceptibility to postoperative cognitive complications [41]. Furthermore, baseline inflammatory status may also play a role, as evidenced by a study from Xiang et al. (2017), which found that elevated postoperative C-reactive protein (CRP) levels were predictive of delirium in elderly patients undergoing colorectal cancer surgery [14].

The patient-level risk factors for POCD in the elderly colorectal cancer patient are complex and additive, with older age, pre-existing impaired cognition, and frailty being the strongest and most consistently demonstrated predictors. These fundamental risk factors are rarely found in isolation, but are instead clustered with low nutritional status, comorbidity, and subclinical cerebrovascular disease to create a synergistic vulnerability profile. While some factors are non-modifiable (age, prior lacunar infarcts), others—such as malnutrition, anemia, and potentially inflammatory status—offer opportunities for preoperative optimization. The practical implication is that risk stratification should be systematic and comprehensive, using validated tools to identify high-risk patients who would benefit from targeted interventions. Critically, patient-level risk factors must be combined with both surgical and anesthetic considerations to create personalized perioperative plans of care that address the heterogeneous nature of cognitive susceptibility.

### 4.2. Surgical and Procedural Factors

Surgical and procedural factors represent a critical category of modifiable risk factors for postoperative cognitive dysfunction in elderly colorectal cancer patients. Unlike patient-related vulnerabilities, which are often fixed or only partially modifiable, surgical and anesthetic management strategies can be directly optimized to reduce cognitive risk. These factors can be conceptually organized into three domains: surgical technique and intraoperative variables, integrated perioperative care pathways, and anesthetic management strategies.

#### 4.2.1. Surgical Technique and Approach

Surgical techniques and factors have long been considered risk factors for POCD, but the evidence is somewhat variable. Longer operative time and more blood loss during operation are generally indicative of worse cognitive outcome, probably the result of greater surgical stress and systemic inflammation [42,43]. Regarding surgical approach, evidence from colorectal surgery suggests that minimally invasive techniques may offer cognitive benefits. A meta-analysis by Lee et al. (2020) found that laparoscopic approaches were associated with lower postoperative delirium rates compared to open surgery in elderly colorectal cancer patients [44]. Ito et al. (2019) also concurred with this finding in their propensity score-matched study of major abdominal surgery, with decreased delirium risk (HR 0.30, *p* = 0.019) with the laparoscopic approach [45]. Jia et al. (2014) had already found that a fast-track program including laparoscopy reduced postoperative delirium from 12.9% to 3.4% in elderly patients undergoing colorectal surgery [17]. These findings suggest that surgical technique optimization, particularly through minimally invasive approaches, may contribute to lower POCD risk, probably reducing tissue trauma, inflammatory responses, and postoperative pain.

#### 4.2.2. Enhanced Recovery After Surgery (ERAS) Protocols

Integrated perioperative care pathways, particularly Enhanced Recovery After Surgery (ERAS) protocols, represent an evidence-based approach to reducing POCD in elderly surgical patients [46,47]. ERAS is a multimodal, multidisciplinary approach to enhancing quick recovery after surgery and inducing a postoperative return to baseline physiology, aiming for the normalization of organ function, by simultaneously optimizing every aspect of perioperative care that may influence rapid recovery; this includes preoperative patient education, goal-directed fluid therapy, multimodal opioid-sparing analgesia, early mobilization, and early nutrition [48]. The efficacy of ERAS in reducing cognitive complications has been consistently demonstrated across multiple studies. The recent meta-analysis of randomized controlled trials by Chen et al. found that ERAS significantly reduced the overall incidence of postoperative delirium (RR = 0.38, *p* < 0.00001), delayed its onset, reduced its duration, and improved cognitive function scores [46]. The American Society for Enhanced Recovery/Perioperative Quality Initiative consensus statement offers a strong recommendation (Grade B) that such multicomponent non-pharmacologic interventions, including ERAS pathways, be employed to prevent postoperative delirium in elderly and high-risk patients [47].

Goal-directed fluid therapy, as one of the essential components of ERAS, has been shown to reduce the risk of POCD in elderly patients undergoing colorectal cancer surgery by maintaining hemodynamic stability and tissue perfusion [49]. Multimodal analgesia protocols and optimized anesthetic techniques as part of the ERAS pathway have shown potential to enhance cognitive outcomes in elderly surgical patients [50]. The randomized controlled trial by Sun et al. (2024) showed that preemptive multimodal analgesia effectively reduced the systemic inflammatory response in patients undergoing laparoscopic colorectal surgery, which is believed to contribute to a lower risk of POCD [13]. The protective actions of ERAS pathways on outcomes not only in colorectal surgery but also in a more generalized GI oncologic tumor patient population [51] further support the applicability of this approach.

#### 4.2.3. Anesthetic Management Strategies

Anesthetic management strategies also play a critical role in modulating cognitive risk. The selection of the analgesic agents, especially the opioids, may affect postoperative cognition. Although opioids are typically required for optimal pain management, high doses elevate the risk of developing delirium [52,53]. The agent used might matter when opioids are necessary; recent evidence indicates that a course of oxycodone may be preferable to one with sufentanil for reducing postoperative delirium in elderly patients undergoing major abdominal surgery [54]. Regional anesthesia techniques, including quadratus lumborum block [55] and paravertebral nerve block [56], have led to significant reductions in POCD incidence in elderly patients undergoing abdominal cancer surgery, likely through reduced systemic opioid requirements and attenuation of the surgical stress response. The choice of anesthetic agents is also an active area of investigation.

In the last few years, newer drugs, such as remimazolam (a short-acting benzodiazepine) and adjuvants such as dexmedetomidine (an α2 agonist with potential neuroprotective effects), have been investigated for their cognitive effects when combined with regional anesthesia techniques [50,57]. However, not all intraoperative variables have an obvious or consistent effect; studies on the impact of intraoperative oxygen concentration on delirium risk, for example, have been conflicting [58], highlighting the complexity of optimizing every aspect of anesthetic management.

In summary, surgical and procedural factors represent the most modifiable domain of POCD risk, providing clinicians with tangible opportunities to prevent cognitive morbidity through evidence-based practice. Minimally invasive approaches, where appropriate oncologically, minimize tissue trauma and inflammatory stimulus. The use of ERAS protocols, which include a collection of evidence-based interventions that collaborate within an integrated perioperative strategy, has consistently demonstrated a decrease in the incidence of POCD and can be considered the standard of care for elderly patients undergoing colorectal cancer surgery. Anesthetic management strategies, particularly multimodal opioid-sparing analgesia and the use of regional anesthesia techniques, further contribute to cognitive protection. Although some aspects, such as the preferred opioid agents and anesthetic adjuvants, need further validation, the general principle is established that proactive, multimodal surgical and anesthetic management with a focus on minimizing surgical stress, optimizing hemodynamic stability, and limiting opioids is crucial in preventing POCD among elders.

Table 2 provides a comprehensive classification of patient-related, surgical, perioperative care, anesthetic, and biological risk factors for POCD, along with their strength of association and corresponding clinical actions for risk mitigation.

## 5. Pathophysiology

The dominant theory on the pathophysiology of POCD is that neuroinflammation serves as a singular end pathway from various perioperative stressors, which ultimately leads to cognitive dysfunction in the susceptible aging brain. Surgical trauma triggers a robust systemic inflammatory response characterized by the release of pro-inflammatory cytokines, such as interleukin-6 (IL-6) and tumor necrosis factor-alpha (TNF-α), into the systemic circulation. In an elderly brain with an impaired blood–brain barrier (BBB), these circulating cytokines can penetrate the BBB and trigger a neuroinflammatory process. A key study by Taylor et al. (2022) provided direct evidence for this mechanism, demonstrating a significant association among postoperative delirium, increased BBB permeability, and elevated cerebrospinal fluid markers of neuroinflammation [6]. This neuroinflammatory condition is characterized by microglial and astrocyte activation, leading to the dysfunction of synaptic plasticity, neurotransmission deficits, and cognitive decline.

The clinical relevance of this inflammatory response is directly applicable to the colorectal cancer surgery population. Studies have shown that interventions that reduce systemic inflammation, such as pre-emptive multimodal analgesia, correlate with better cognitive outcomes in this specific cohort [13]. In addition, inflammatory biomarkers help predict delirium risk in colorectal surgery. Preoperative C-reactive protein levels have been validated as independent predictors of POD in abdominal surgery [14], and novel biomarkers, such as serum microRNA-155, are promising for the early identification of high-risk patients who may develop cognitive dysfunction after colorectal cancer surgery [18]. These results highlight the value of inflammatory biomarkers for risk stratification and targeted preventive interventions.

In addition to the general mechanisms of systemic inflammation, colorectal surgery presents a unique and clinically relevant pathophysiologic mechanism through disruption of the gut–brain axis. Surgical manipulation of the bowel, including perioperative antibiotic use and the inflammatory response, contributes to significant changes in the gut microbiome, known as dysbiosis. Recent evidence indicates that this dysbiosis is not a result of surgery per se but may itself contribute to neuroinflammation and resultant cognitive deterioration. A clinical study by Zhang et al. (2023) demonstrated an association between postoperative gut microbiota composition, specifically Parabacteroides distasonis, and postoperative delirium in elderly patients undergoing orthopedic surgery [59]. Furthermore, experimental evidence from Zhang et al. (2019) showed that fecal microbiota transplantation from mice that developed delirium-like behaviors after abdominal surgery was sufficient to induce similar behavioral impairments in recipient mice, establishing a potential direct mechanistic connection between gut dysbiosis and cognitive dysfunction [60].

**Figure 1:**
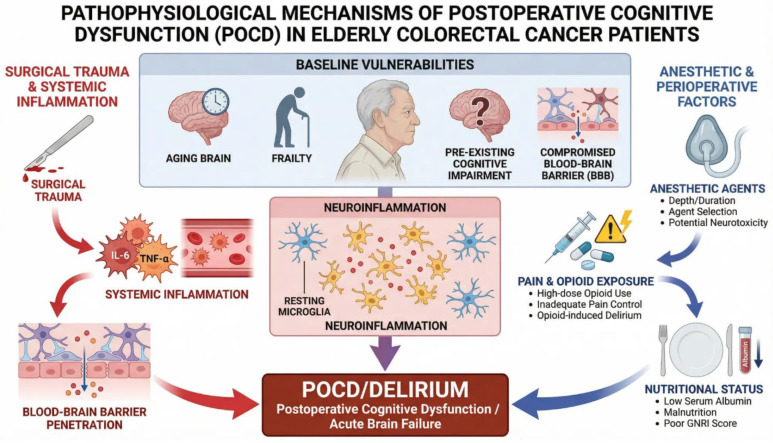
Pathophysiological mechanisms of postoperative cognitive dysfunction (POCD) in elderly colorectal cancer patients. Baseline vulnerabilities in the aging brain include preoperative cognitive impairment, frailty, and compromised blood–brain barrier integrity. Surgical trauma triggers systemic inflammation with elevated pro-inflammatory cytokines (IL-6, TNF-alpha, IL-1beta), which penetrate the BBB and activate microglial cells. Anesthetic and perioperative factors contribute through direct neurotoxic effects, pain-related opioid exposure, and poor nutritional status. These pathways converge on neuroinflammation, leading to POCD and delirium.

These findings collectively support a unified pathophysiological model in which neuroinflammation is the final common pathway for POCD, and colorectal surgery operates as a unique “double-hit” mechanism. The first hit derives from the systemic inflammatory response to surgical trauma, common to all major surgeries. The second, more specific hit arises from disruption of the gut–brain axis, whereby surgical manipulation and dysbiosis amplify neuroinflammatory signaling via microbial metabolites and immune mediators. This dual mechanism could account for why patients undergoing colorectal surgery display specific susceptibility to cognitive disturbances and how conventional anti-inflammatory approaches may be inadequate. The model also suggests several therapeutic targets, including perioperative maintenance of the microbiome through selective antibiotic stewardship, probiotic or prebiotic supplementation, optimization of systemic anti-inflammatory strategies, and protection of the blood–brain barrier. Understanding these interconnected pathways is essential for developing comprehensive, mechanistically informed prevention strategies tailored to the unique pathophysiology of POCD in colorectal cancer surgery (Figure 1).

## 6. Prevention and Management

### 6.1. Preoperative Risk Assessment and Optimization

#### 6.1.1. Cognitive Screening and Frailty Assessment

The prevention of POCD in elderly colorectal cancer patients begins with a comprehensive preoperative risk assessment. Preoperative cognitive screening using validated instruments is a critical first step in risk stratification because baseline cognitive impairment is one of the strongest predictors of postoperative delirium. Jones et al. (2016) demonstrated in a prospective cohort study that the risk of delirium is linearly and strongly associated with presurgical cognitive performance, even at levels above the population median [61]. The Montreal Cognitive Assessment (MoCA) and Mini-Mental State Examination (MMSE) are the most commonly used screening tools in the perioperative setting. Eschweiler et al. (2021) demonstrated in a prospective study of 880 older patients undergoing elective surgery that a clinical–cognitive model incorporating MoCA scores, along with ASA classification and expected surgery duration, predicted postoperative delirium risk with high accuracy (AUC = 0.80; 95% CI 0.76–0.84) [62]. Yang et al. (2020) demonstrated that a preoperative MMSE score less than 24 was strongly associated with an increased risk of POCD [9].

Frailty assessment is equally critical in preoperative risk stratification. Frailty, characterized by a state of increased susceptibility to stressors as a result of diminished physiological reserves, is a more effective indicator of postoperative outcomes than chronological age alone. A systematic review by Fagard et al. (2016) demonstrated that frail patients undergoing elective colorectal cancer surgery had significantly higher risks of moderate to severe postoperative complications, longer hospital stays, higher readmission rates, and decreased long-term survival compared to non-frail patients [63]. Recognizing the importance of frailty assessment, the American Society of Colon and Rectal Surgeons (ASCRS) published comprehensive clinical practice guidelines in 2022 recommending that frailty assessment, rather than chronological age alone, should guide surgical decision making in older adults undergoing colorectal surgery [64]. The Modified Frailty Index (mFI) and Clinical Frailty Scale (CFS) are among the most widely used screening tools in perioperative environments because of their simplicity and proven predictive accuracy. For frail patients, a geriatric assessment could be used for perioperative optimization.

#### 6.1.2. Medication Review and Polypharmacy Management

The preoperative optimization of medication is a critical but often neglected aspect of POCD prevention. Polypharmacy and a high anticholinergic burden are both known risk factors for POCD. Anticholinergics, such as diphenhydramine, oxybutynin, and some antidepressants, can affect cholinergic neurotransmission by crossing the blood–brain barrier, thereby increasing the risk of delirium. A systematic review by Egberts et al. (2021) found consistent evidence that the anticholinergic drug burden, as measured using the Anticholinergic Risk Scale (ARS), was associated with an increased risk of delirium across multiple studies [65]. Similarly, benzodiazepines, antipsychotics, and other potentially deliriogenic drugs should be examined and minimized if possible. Duprey et al. (2022) demonstrated that postoperative benzodiazepine administration was associated with a 3-fold increased risk of delirium in older adults undergoing elective non-cardiac surgery (aHR 3.23, 95% CI 2.10–4.99) [66]. Preoperative medication reviews should focus on deprescribing unnecessary anticholinergic medications, reducing polypharmacy, and optimizing the management of chronic conditions without introducing additional cognitive risk. The ASCRS guidelines suggest that a comprehensive medication review should be performed as part of preoperative assessment for colon and rectal surgery in the elderly [64].

#### 6.1.3. Prehabilitation and Patient Education

Prehabilitation, a multimodal preoperative intervention to improve functional capacity and physiological reserve, has been proposed as an effective approach in this context for reducing postoperative complications and cognitive dysfunction. Multimodal prehabilitation programs typically combine exercise training, nutritional optimization, and psychological support. The ASCRS guidelines support preoperative multimodal optimization (prehabilitation) for frail elderly patients who are undergoing colorectal resection. A landmark randomized controlled trial by Barberan-Garcia et al. (2018) in high-risk patients (age > 70 years and/or ASA III/IV) undergoing elective major abdominal surgery demonstrated that personalized prehabilitation reduced the proportion of patients experiencing postoperative complications by 51% (RR 0.5; 95% CI 0.3–0.8, *p* = 0.001) [67]. Specifically for colorectal surgery, a multicenter randomized controlled trial by Berkel et al. (2022) demonstrated that a 3-week community-based exercise prehabilitation program reduced 30-day postoperative complications in high-risk patients scheduled for elective colon resection (RR 0.59; 95% CI 0.37–0.96, *p* = 0.024) [68]. While direct evidence linking prehabilitation to reduced POCD in colorectal surgery is still emerging, the mechanistic rationale is strong: improved physiological reserve, reduced systemic inflammation, and enhanced stress resilience may all contribute to cognitive protection.

Patient and family education represents a critical but often underutilized component of preoperative preparation. Patients and families should be informed about the risk of postoperative delirium, its presentation, and preventive factors to promote partnerships in preventive measures and in recognizing symptoms. The ASER/POQI consensus statement emphasizes the importance of patient and family education as part of a multicomponent delirium prevention strategy [47]. Structured preoperative discussions that engage family members and interdisciplinary teams can improve postoperative outcomes by ensuring that older patients and their caregivers have a realistic understanding of surgical risks, recovery expectations, and the importance of delirium prevention strategies [69]. Shared decision-making around discussing cognitive risk is particularly relevant for older people with pre-existing dementia risk or frailty, who will also need to carefully weigh the potential surgical benefit compared with the risk of translation into cognitive decline.

In conclusion, systemic preoperative evaluation and optimization are cornerstones for preventing POCD in older CRC patients. This may involve structured cognitive and frailty assessment to identify individuals at high risk, medication review to minimize the anticholinergic and deliriogenic load, prehabilitation to optimize physiological reserve, and patient empowerment to involve them in shared decision making. These evidence-based preoperative strategies, if used routinely, can reduce the prevalence of postoperative cognitive dysfunction and improve overall surgical outcomes.

### 6.2. Intraoperative and Anesthetic Strategies

#### 6.2.1. Multimodal Opioid-Sparing Analgesia

Effective intraoperative anesthetic and analgesic management is pivotal in reducing postoperative cognitive dysfunction, as the decisions taken concerning anesthetics directly correlate with neuroinflammation, the management of pain, and the overall stress response to surgery. Multimodal opioid-sparing analgesia plays a central role in neuroprotective perioperative care by combining regional anesthesia methods with non-opioid adjuncts to reduce opioid requirement while ensuring appropriate pain relief. A recent randomized controlled trial in laparoscopic colorectal surgery demonstrated that pre-emptive multimodal analgesia significantly reduced the systemic inflammatory response, which is a key driver of postoperative cognitive complications [13]. Regional anesthesia techniques, such as the transversus abdominis plane (TAP) block, not only provide superior analgesia but may also contribute to cognitive protection by reducing opioid requirements and modulating the surgical stress response. Zhang et al. (2025) reported an association between the postoperative use of dexmedetomidine and TAP block, leading to improved recovery following CRC surgery, suggesting that combined administration might exhibit synergistic neuroprotective properties [57].

#### 6.2.2. Anesthetic Agent Selection

The selection of primary anesthetic agents has emerged as an essential consideration for cognitive protection in elderly surgical patients. Classical agents, including propofol and inhaled anesthetics, have been the mainstay for this purpose; however, newer agents with potential for improved cognitive profile are being explored. Recently, Remimazolam, a new ultra-short benzodiazepine anesthesia induction agent, has been known for its neuroprotective effects. A dose-finding study suggested that moderate-dose Remimazolam (1.0 mg/kg/h) may offer optimal cognitive protection compared to low-dose Remimazolam or propofol in elderly colorectal cancer patients [70]. Dexmedetomidine, an α2-adrenergic agonist with sedative, analgesic, and anti-inflammatory properties, is increasingly recognized as an efficient adjuvant neuroprotective agent in colorectal surgical procedures. Its hemodynamic stability, opioid-sparing effects, and ability to decrease delirium incidence make it an appealing alternative in high-risk elderly patients [57,71].

In addition to classical anesthetics, several new pharmacologic agents are under investigation for protecting cognitive function during colorectal procedures. Systemic lidocaine infusions have been investigated for their anti-inflammatory, analgesic, and potential neuroprotective effects, with ongoing trials exploring their role in reducing postoperative cognitive complications [72]. Ketamine, traditionally used for anesthesia and analgesia, has demonstrated neuroprotective properties through its modulation of glutamatergic neurotransmission and anti-inflammatory effects. Also, melatonin, a neurohormone with antioxidant and anti-inflammatory properties, is feasible for preventing postoperative delirium, as shown in a recent randomized controlled trial in patients undergoing colorectal cancer surgery [73]. These interventions are promising, although additional large-scale investigations are required to determine their efficacy and optimal timing for preventing cognitive decline in elderly colorectal surgery patients.

#### 6.2.3. Non-Pharmacological Strategies

Non-pharmacological intraoperative strategies represent an innovative complementary approach to cognitive protection. Transcutaneous electrical acupoint stimulation (TEAS), a technique derived from traditional Chinese medicine, has demonstrated potential to reduce postoperative cognitive dysfunction by modulating inflammatory markers and the gut–brain axis [74]. Such a strategy is particularly valuable in colorectal surgery, as manipulation of the bowel and subsequent alteration of the gut microbiome may contribute to peripheral mechanisms of neuroinflammation and cognitive impairment. TEAS offers the advantage of being non-invasive, devoid of pharmacological side effects, and potentially synergistic with other neuroprotective strategies. Although the literature on TEAS is still growing, available evidence suggests that it could be a valuable adjunct to multimodal cognitive protection strategies.

In summary, multimodal opioid-sparing analgesia, including the use of regional anesthetic techniques when appropriate, should remain a central theme in the neuroprotective strategy for elderly patients undergoing colorectal cancer surgery. The choice of anesthetic agents should consider emerging evidence favoring agents with neuroprotective properties, such as dexmedetomidine as an adjuvant and potentially Remimazolam as a primary agent. Several classes of new drugs, such as systemic lidocaine, ketamine, and melatonin, have shown promising results in preliminary clinical trials and deserve further testing in large randomized prospective studies. Non-drug-based treatments, including TEAS, may provide an adjunctive treatment, particularly in colorectal surgery, where gut–brain axis function is involved. The active research in this field reflects the growing recognition that anesthetic management is not merely about maintaining unconsciousness and analgesia but represents a critical opportunity to modulate the surgical stress response and protect cognitive function in vulnerable elderly patients.

### 6.3. Postoperative Care

Postoperative care represents a critical window for preventing and managing cognitive dysfunction in elderly colorectal cancer patients. The cornerstone of delirium prevention in this phase is the implementation of structured, multicomponent, non-pharmacologic intervention bundles. These evidence-based bundles typically address multiple modifiable risk factors simultaneously, including cognitive stimulation through orientation and therapeutic activities; sleep promotion through noise reduction and circadian rhythm maintenance; early mobilization to prevent functional decline; adequate hydration and nutrition; and management of sensory impairments through the provision of vision and hearing aids. The multicomponent approach recognizes that postoperative delirium is multifactorial in etiology and therefore requires multifaceted prevention strategies that address the complex interplay of physiological, environmental, and iatrogenic risk factors.

#### 6.3.1. Multicomponent Non-Pharmacologic Interventions

Multicomponent non-pharmacologic interventions are effective across a wide range of clinical settings. The landmark Hospital Elder Life Program (HELP), developed by Inouye and colleagues, has demonstrated significant reductions in delirium incidence in hospitalized older adults, including those in surgical intensive care units [75]. Recognizing the specific needs of surgical populations, adaptations of HELP have been developed and validated. The t-HELP was explicitly designed for surgical patients and is equally effective as the standard HELP in preventing postoperative delirium [76]. Chen et al. (2017) further validated a modified HELP protocol adapted for surgical populations, demonstrating significant reductions in delirium incidence and length of stay through systematic implementation of multicomponent non-pharmacological interventions, including cognitive stimulation, early mobilization, and sleep hygiene [77]. The principles of these multicomponent interventions have been successfully applied to the colorectal surgery population. In colorectal surgery specifically, a meta-analysis demonstrated that enhanced-recovery protocols reduced the incidence of delirium by 55% (RR 0.45; 95% CI: 0.21–0.98) [78].

#### 6.3.2. Pain Management and Early Mobilization

Apart from organized multicompound programs, there are several specific issues in postoperative management that could be further highlighted in terms of cognitive protection. There is a need to connect with adequate pain control and opioid-sparing techniques, including multimodal analgesia that begins intraoperatively and continues into post-operative management [53]. Early mobilization is strongly promoted as a cornerstone of Enhanced Recovery After Surgery (ERAS) pathways. It serves multiple purposes: preventing functional decline, reducing the risk of complications such as pneumonia and thromboembolism, and providing cognitive stimulation through physical activity [48]. It is necessary to conduct routine screening for delirium using a validated tool, such as the Confusion Assessment Method (CAM), to facilitate early detection and alert clinicians to the need for intervention should delirium develop despite preventive strategies [79]. Regular delirium screening should be incorporated into routine nursing assessments, particularly during the first 72 h postoperatively when delirium risk is highest [80].

Adequate postoperative cognitive protection in elderly colorectal cancer patients requires a systematic, multicomponent approach addressing the multifactorial nature of delirium. Evidence-based programs such as HELP and its surgical adaptations provide validated frameworks with demonstrated efficacy in colorectal surgery populations. Success depends on coordinated implementation by multidisciplinary teams, recognizing that delirium prevention is not the responsibility of a single clinician but requires integrated efforts across the entire perioperative care team. Healthcare systems should establish structured multicomponent delirium prevention protocols as standard care, with ongoing monitoring to ensure sustained effectiveness.

## 7. Future Directions

Although much progress has been made regarding the prevention and knowledge of POCD in elderly patients with colorectal cancer, significant knowledge gaps remain. Future research must address several critical areas to optimize cognitive outcomes in this vulnerable population. These include developing robust predictive models for individualized risk stratification, validating novel neuroprotective interventions, and clarifying optimal perioperative management strategies. Below, we articulate major research priorities that have the potential to move the field forward.

The development of robust, multimodal predictive models represents a high priority for future research. Huang et al. (2022) developed and validated a nomogram incorporating multiple clinical and laboratory predictors (age, MMSE, albumin, surgical duration, IL-6) for predicting POCD in elderly patients undergoing gastrointestinal tumor resection, achieving an AUC of 0.85 and providing a practical clinical tool for individualized risk assessment [81]. While such models show promise, they require external validation in larger, diverse cohorts.

The discovery and validation of novel, more specific biomarkers of neuronal injury and neuroinflammation that can be measured non-invasively (e.g., in blood) remains essential. Advanced neuroimaging techniques, such as high-field 7T MRI, have revealed specific brain network alterations associated with cognitive dysfunction in colorectal cancer patients, offering potential for the early detection and monitoring of at-risk individuals [60]. Machine learning models incorporating several blood biomarkers, such as lipid markers and TMAO, have shown encouraging efficacy for POCD prediction in CRC surgery, representing a major advance toward personalized risk stratification [82].

Non-pharmacological neuroprotective approaches should be further studied as safe and cost-effective interventions. Remote ischemic preconditioning, an easy preoperative procedure involving repeated brief periods of limb ischemia–reperfusion, was found to be effective in a recent RCT by He et al. (2017), enhancing postoperative cognitive function and reducing levels of inflammatory markers after colon surgery in elderly individuals [83]. However, larger multicenter trials are needed to establish its clinical utility [83]. Similarly, transcranial direct current stimulation (tDCS), a non-invasive brain stimulation technique, demonstrated a significant reduction in postoperative delirium incidence from 25.5% to 8.2% (*p* < 0.001) in a recent RCT by Li et al. (2024) in patients undergoing laparoscopic colorectal cancer surgery, which is another potential approach to non-pharmacologic neuroprotection [84]. These approaches should be confirmed in further large, multicenter studies before they can be considered for clinical application.

The optimal anesthetic and pharmacological approach for cognitive protection remains under investigation. While the benefits of multimodal, opioid-sparing anesthesia are clear, the optimal combination of agents and techniques requires clarification through large-scale comparative effectiveness trials. Specific agents such as dexmedetomidine, ketamine, and lidocaine need further evaluation to define the ideal anesthetic regimen for cognitive protection in this high-risk population [72]. Future trials of innovative anesthetics, such as xenon in colorectal surgery, may provide crucial evidence on neuroprotective strategies [85]. Novel pharmacological interventions targeting neuroinflammation are also under investigation. Zhang et al. (2024) demonstrated in a double-blind pilot RCT that intranasal insulin significantly reduced the POCD incidence from 38.7% to 13.3% (*p* = 0.024) in elderly patients undergoing laparoscopic colorectal cancer surgery, alongside reductions in the inflammatory markers IL-6, TNF-α, and S100β, highlighting a possible avenue for neuroprotection [86]. Duan et al. (2021) performed a meta-analysis and showed that ulinastatin, a broad-spectrum protease inhibitor with anti-inflammatory effects, significantly decreased POCD incidence among elderly surgical patients by alleviating systemic inflammation and cytokine release, supporting the rationality of applying anti-inflammatory strategies for cognitive protection [87].

**Figure 2:**
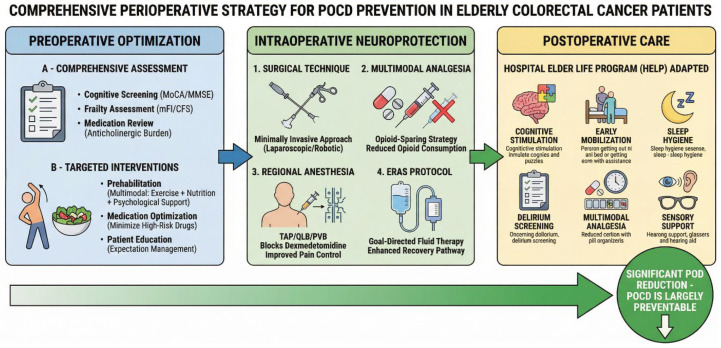
Comprehensive perioperative strategy for POCD prevention in elderly colorectal cancer patients. Preoperative optimization includes cognitive and frailty assessment, medication review, multimodal prehabilitation, and patient education. Intraoperative neuroprotection employs minimally invasive techniques, multimodal opioid-sparing analgesia, regional anesthesia with dexmedetomidine, and ERAS protocols. Postoperative care implements an adapted HELP bundle with cognitive stimulation, early mobilization, sleep hygiene, delirium screening, multimodal analgesia, and sensory support. This integrated approach achieves a significant reduction in postoperative delirium, demonstrating that POCD is largely preventable.

The future of cognition protection among elderly colorectal cancer patients rests on a multifaceted approach including risk prediction, targeted interventions, and personalized care in perioperative settings. Priority should be given to large-scale, multicenter trials validating promising interventions such as remote ischemic preconditioning and transcranial direct current stimulation, as well as comparative effectiveness studies defining optimal anesthetic protocols. Incorporating advanced biomarkers, machine learning algorithms, and neuroimaging into the clinical setting may offer hope of identifying high-risk individuals who will benefit most from aggressive preventive strategies. Finally, implementing these research findings into established clinical practice will be a team effort among surgeons, anesthesiologists, geriatricians, and neuroscientists working within health systems that prioritize cognitive outcomes as a significant quality target for the elderly undergoing surgical procedures (Figure 2).

A significant evolution in perioperative neurocognitive research involves shifting the focus from merely identifying risk factors to understanding the mechanisms of cognitive protection and recovery. Future investigations should therefore explore the concepts of cognitive reserve and cognitive resilience [88]. Cognitive reserve refers to the brain’s passive capacity to withstand pathological insults, and a higher reserve—often associated with lifelong intellectual enrichment—has been consistently linked to a lower risk of postoperative neurocognitive disorders [88,89]. Beyond this static concept, cognitive resilience represents the active, dynamic process of adapting to and recovering from the surgical stressor [90]. This framework could be further expanded to include psychological resilience, as patients’ coping mechanisms and mental fortitude have also been shown to positively influence postoperative recovery trajectories [91,92]. Adopting this perspective, future longitudinal studies could employ advanced statistical models, such as latent class analyses, to identify distinct phenotypes of cognitive recovery. This would allow the development of targeted interventions, such as preoperative cognitive training or psychological support, to bolster patient resilience before surgery.

## 8. Limitations of This Review

The authors acknowledge several limitations inherent to this review’s methodology. First, as a narrative review, its primary goal is to provide a comprehensive overview and synthesis of the topic from the authors’ perspective, rather than to answer a pre-defined clinical question through an exhaustive and reproducible search protocol, such as PRISMA. While this approach allows for a broad and integrated discussion, it is distinct from a systematic review, and therefore, the selection of studies is inherently tailored to the narrative’s scope and may not encompass every relevant article on the subject. Second, the review provides a qualitative synthesis of the evidence, and no formal meta-analysis was performed to quantitatively assess the magnitude of the effects reported. Finally, a formal risk-of-bias assessment of the included studies using standardized tools was not conducted. Beyond these methodological considerations inherent to the review’s format, a comprehensive perspective also requires a critical appraisal of the current evidence base itself, particularly concerning the foundational studies for the main interventions discussed. Furthermore, while this review highlights several promising interventions, the strength of the supporting evidence warrants careful consideration. Foundational evidence for key strategies such as ERAS protocols [17] and multicomponent HELP interventions [77] often originates from single-center or cluster-randomized trials, respectively. These designs, while pragmatic, may limit the generalizability of the reported effect sizes. Likewise, emerging concepts such as the role of the gut–brain axis are supported primarily by pre-clinical models [60] and observational human studies [59], which establish association but not causation. Similarly, many of the novel neuroprotective strategies discussed—including remote ischemic preconditioning [83], transcranial direct current stimulation [84], and intranasal insulin [86]—are supported by pilot or exploratory RCTs with small sample sizes. Although these studies are crucial for driving innovation, their findings should be interpreted as preliminary until they are validated by larger, adequately powered, multicenter trials.

## 9. Conclusions

Postoperative cognitive dysfunction represents a predictable and potentially preventable complication in elderly patients undergoing colorectal cancer surgery. The evidence synthesized in this review demonstrates that POCD arises from a complex interplay between patient-specific vulnerabilities and perioperative stressors, with incidence rates ranging from 3.4% in optimized care pathways to 35% in high-risk cohorts.

Effective management begins with proactive risk stratification. Advanced age, preoperative cognitive impairment, and frailty consistently emerge as the strongest predictors of POCD. The use of validated screening tools such as the Mini-Mental State Examination (MMSE), Montreal Cognitive Assessment (MoCA), and comprehensive geriatric assessment (CGA) in routine clinical practice allows for the early identification of high-risk individuals who may benefit from targeted preoperative optimization, including nutritional support, prehabilitation, and medication review to reduce the anticholinergic burden.

Perioperative management strategies represent powerful modifiable factors in mitigating POCD risk. Enhanced Recovery After Surgery (ERAS) protocols, incorporating minimally invasive surgical techniques, multimodal opioid-sparing analgesia, and early mobilization, have demonstrated substantial efficacy. After surgery, multicomponent, nondrug intervention bundles such as the Hospital Elder Life Program (HELP) and its surgical models offer an evidence-based mechanism for systematic delirium prevention by implementing comprehensive attention to cognitive stimulation, sleep hygiene, hydration, and early mobilization.

The pathophysiology of POCD is increasingly understood through the lens of neuroinflammation triggered by systemic surgical stress. Emerging research into the gut–brain axis has revealed that surgery-induced dysbiosis may amplify this neuroinflammatory cascade, particularly relevant in colorectal surgery, where direct bowel manipulation occurs. This mechanistic insight opens novel therapeutic avenues, including microbiome-targeted interventions, neuroprotective strategies such as intranasal insulin, and anti-inflammatory approaches currently under investigation.

Despite these advances, significant knowledge gaps remain. Robust, externally validated predictive models incorporating clinical and biomarker data are needed to refine risk stratification. The optimal sequencing and combination of pharmacological neuroprotective agents require further investigation through adequately powered randomized controlled trials. Future studies must also investigate cognitive trajectories in elderly patients with CRC and interventions that not only mitigate acute delirium but also contribute to its long-term prevention and safeguard current cognition. Mitigating the cognitive impact of colorectal cancer surgery in the elderly demands a paradigm shift toward proactive, multidisciplinary perioperative care that seamlessly integrates geriatric principles across the entire surgical pathway—from preoperative assessment and optimization through tailored intraoperative management to structured postoperative care. By systematically addressing patient vulnerability while minimizing perioperative insults, it is possible to significantly reduce the burden of POCD and improve outcomes for this growing patient population.

## Figures and Tables

**Table 1:** Assessment tools for POCD risk stratification and delirium detection in elderly colorectal cancer patients

Assessment Domain	Tool Name	Cutoff/Criteria	Clinical Application	Validated Outcomes
Preoperative cognitive screening	MMSE (Mini-Mental State Examination)	<24 points indicates cognitive impairment and increased POCD risk.	A bedside cognitive screening tool assessing orientation, memory, attention, language, and visuospatial function. Validated predictors of POCD in CRC surgery populations.	MMSE < 24 is significantly associated with increased POCD risk in a meta-analysis of patients undergoing CRC surgery. Simple, widely available tool for baseline cognitive assessment.
Preoperative cognitive screening	MoCA (Montreal Cognitive Assessment)	<26 points indicates cognitive impairment (standard cutoff); more sensitive than MMSE for mild deficits.	Comprehensive cognitive screening tool assessing multiple domains, including executive function, attention, memory, language, and visuospatial abilities. Recommended for preoperative evaluation when higher sensitivity is desired.	Superior sensitivity for detecting mild cognitive impairment compared to MMSE. When incorporated in a multivariable model with ASA classification and surgery duration, it achieves high predictive accuracy for POD (AUC 0.80, 95% CI 0.76–0.84) in a prospective cohort of 880 elderly surgical patients.
Preoperative frailty assessment	mFI (modified Frailty Index)	Higher scores (range 0–1) predict worse postoperative outcomes; no universal cutoff, but scores >0.27 often indicate frailty.	Simple 11-item frailty screening tool based on comorbidities and functional status. It can be calculated from routine preoperative data and validated in surgical populations, including CRC.	Frail patients have significantly higher postoperative complications, longer hospital stays, higher readmission rates, and decreased long-term survival. ASCRS recommends frailty assessment over chronological age alone for surgical decision making.
Preoperative frailty assessment	CFS (Clinical Frailty Scale)	Scores 1–9: 1 = very fit, 2 = well, 3 = managing well, 4 = vulnerable, 5 = mildly frail, 6 = moderately frail, 7 = severely frail, 8 = very severely frail, 9 = terminally ill. Scores ≥5 indicate frailty.	Visual-based frailty assessment using pictorial representations and clinical descriptors. Rapid bedside tool requiring no special equipment. Validated across multiple surgical settings.	Predicts postoperative complications, mortality, and functional decline. Scores ≥5 indicate that patients require a comprehensive geriatric assessment and intensive perioperative optimization—strong correlation with adverse outcomes in elderly surgical patients.
Preoperative nutritional screening	GNRI (Geriatric Nutritional Risk Index)	Calculated as [1.489 × albumin (g/L)] + [41.7 × (current weight/ideal weight)]. Lower scores indicate higher nutritional risk.	Simple nutritional screening tool using readily available laboratory (albumin) and anthropometric (weight) data. Designed explicitly for elderly populations.	Validated predictor of postoperative complications, including delirium, in elderly CRC patients. Low GNRI scores are associated with increased POD risk, longer hospital stays, and higher morbidity. Identifies patients requiring preoperative nutritional optimization.
Preoperative medication review	ARS (Anticholinergic Risk Scale)	Scores medications as 0 (no anticholinergic activity), 1 (moderate), 2 (strong), or 3 (very strong). Higher cumulative scores indicate greater anticholinergic burden and delirium risk.	Systematic tool for quantifying anticholinergic burden from all medications. Identifies potentially deliriogenic drugs requiring deprescribing or dose reduction. Guides preoperative medication optimization.	Systematic review demonstrates a consistent association between higher ARS scores and increased delirium risk across multiple studies. Anticholinergic burden is a modifiable risk factor; medication review and deprescribing reduce the incidence of POCDs.
Postoperative delirium detection	CAM (Confusion Assessment Method)	Requires presence of: (1) acute onset and fluctuating course, (2) inattention, and either (3) disorganized thinking or (4) altered level of consciousness.	Gold standard bedside tool for delirium detection. Should be administered systematically during the first 72 h postoperatively when delirium risk is highest—brief (2–5 min), structured assessment.	High sensitivity and specificity for delirium detection when administered by trained personnel. Enables early detection and prompt intervention. Widely validated across surgical populations, including elderly CRC patients.

Legend: ARS, Anticholinergic Risk Scale; ASCRS, American Society of Colon and Rectal Surgeons; AUC, area under the curve; CAM, Confusion Assessment Method; CFS, Clinical Frailty Scale; CI, confidence interval; CRC, colorectal cancer; GNRI, Geriatric Nutritional Risk Index; mFI, modified Frailty Index; MMSE, Mini-Mental State Examination; MoCA, Montreal Cognitive Assessment; POD, postoperative delirium; POCD, postoperative cognitive dysfunction.

**Table 2:** Risk factors for POCD in elderly colorectal cancer patients: classification, strength of association, and clinical actions.

Risk Factor Category	Specific Risk Factor	Strength of Association	Key Clinical Action
Demographic and Clinical	Age > 70 years	Consistent independent predictors across studies	Age-adjusted perioperative care protocols; heightened surveillance
	MMSE < 24	Significant predictor in meta-analysis	Preoperative cognitive screening; tailored interventions for impaired patients
	ASA classification ≥III	Strong association with increased POCD risk	Optimize medical comorbidities preoperatively; consider intensive monitoring
	Multiple comorbidities (cardiovascular, cerebrovascular, diabetes)	Cumulative risk effect	Comprehensive preoperative assessment; multidisciplinary optimization
	History of stroke or TIA	OR 2.5–3.0 for POD	Neurological consultation; optimize cerebrovascular protection strategies
	Pre-existing cognitive impairment	41% POD incidence vs. 11% in normal cognition	Mandatory cognitive screening; intensive preventive measures
	Low educational level	Independent risk factor	Adapted communication strategies; enhanced patient education
Frailty and Functional Status	Cognitive frailty (combined physical + cognitive impairment)	OR 12.86 (strongest predictor identified)	Prehabilitation programs; comprehensive geriatric assessment
	Physical frailty (CFS ≥ 5, mFI > 0.27)	Significant predictor across multiple studies	Frailty-directed interventions; consider frailty as a contraindication to aggressive surgery in some cases
	Sarcopenia	Associated with increased POCD risk	Nutritional support; resistance exercise programs
	Low grip strength	Functional predictors of postoperative complications	Simple bedside assessment; prehabilitation target
Nutritional Status	Low serum albumin (<35 g/L)	Consistent risk factor across studies	Preoperative nutritional optimization: albumin supplementation when indicated
	Low GNRI score	Validated predictor in CRC populations	Nutritional screening; targeted nutritional support
	Malnutrition	Independent risk factor	Dietitian consultation; enteral/parenteral nutrition when appropriate
Surgical Context	Emergency surgery	3.4× higher POD risk vs. elective	Unavoidable, but recognize extreme high-risk status; maximize other protective factors
	Laparoscopic surgery	HR 0.30 for POCD (protective)	Preferred surgical approach for cognitive protection
	Prolonged operative time (>4 h)	Dose–response relationship	Optimize surgical efficiency; avoid unnecessary prolongation
Perioperative Care Pathway	ERAS protocol implementation	RR 0.38 for POD (highly protective)	Implement a comprehensive ERAS pathway as the standard of care.
	Fast-track protocol	73.7% reduction in POCD incidence	Aggressive implementation of fast-track elements
Anesthetic Factors	High-dose opioid use	Strong association with increased delirium risk	Multimodal opioid-sparing analgesia; minimizes opioid exposure
	Lack of regional anesthesia	Missed opportunity for neuroprotection	Implement regional techniques (TAP, QLB, PVB) when feasible
	Prolonged anesthetic depth	Associated with increased POCD risk	BIS monitoring; avoid excessive anesthetic depth
	Use of benzodiazepines	Deliriogenic medication class	Avoid benzodiazepines perioperatively; consider dexmedetomidine as an alternative
Biological Markers	Elevated inflammatory markers (IL-6, CRP)	Mechanistic link to neuroinflammation	Minimize surgical trauma; anti-inflammatory strategies
	Presence of lacunar infarcts on imaging	Structural brain vulnerability	Brain MRI when indicated; recognize increased susceptibility

Legend: ASA, American Society of Anesthesiologists; BIS, Bispectral Index; CFS, Clinical Frailty Scale; CRC, colorectal cancer; CRP, C-reactive protein; ERAS, Enhanced Recovery After Surgery; GNRI, Geriatric Nutritional Risk Index; HR, hazard ratio; IL-6, interleukin-6; mFI, modified Frailty Index; MMSE, Mini-Mental State Examination; OR, odds ratio; POD, postoperative delirium; POCD, postoperative cognitive dysfunction; PVB, paravertebral block; QLB, quadratus lumborum block; RR, relative risk; TAP, transversus abdominis plane; TIA, transient ischemic attack.

## Data Availability

No new data were created or analyzed in this study. Data sharing does not apply to this article.
